# Family refusal of skin donation for transplantation: trends and associated factors

**DOI:** 10.1590/0034-7167-2023-0209

**Published:** 2024-07-29

**Authors:** Karoline de Oliveira Lins Souto, Rafael Rodrigo da Silva Pimentel, Ágata Nunes Brito, Edvaldo Leal de Moraes, Marcelo José dos Santos

**Affiliations:** I Universidade de São Paulo. São Paulo, São Paulo, Brazil; II Universidade de São Paulo, Hospital das Clínicas, Organização de Procura de Órgãos. São Paulo, São Paulo, Brazil

**Keywords:** Skin Donation, Tissue Bank, Family Refusal, Public Health Nursing, Nursing, Donación de Piel, Banco de Tejidos, Rechazo Familiar, Enfermería de Salud Pública, Enfermería

## Abstract

**Objectives:**

to analyze the trends and factors associated with family refusal of skin donation for transplantation.

**Methods:**

this cross-sectional study was conducted in the State of São Paulo, with family authorization terms collected from 2001 to 2020. The variables analyzed included year, age, gender, cause of death, and type of institution. Data were analyzed using linear and multiple logistic regression, with the Odds Ratio estimated at p<0.05 for statistical significance.

**Results:**

1,355 individuals refused skin donation. The trend of refusals decreased between 2001 and 2009 in the age groups of 0-11 years and 12-19 years, but increased in the group aged ≥60 years. This trend continued to decrease in the 0-11 years group from 2010 to 2020, and increased in the 20-40 years group. Males and the age groups of 20-40 years, 41-59 years, and ≥60 years exhibited 27%, 34%, 47%, and 53% lower chances of refusal, respectively.

**Conclusions:**

there is an urgent need for measures to mitigate the high number of refusals associated with skin donation.

## RESUMEN

**Objetivos:**

analizar la tendencia y los factores asociados al rechazo familiar de la donación de piel para trasplante.

**Métodos:**

estudio transversal desarrollado en el Estado de São Paulo con los términos de autorización familiar firmados entre 2001 y 2020. Las variables analizadas fueron: año, edad, sexo, causa del deceso y tipo de institución. Los datos fueron analizados mediante regresión lineal y logística múltiple, con la estimación del Odds Ratio, adoptando p<0,05 como significancia estadística.

**Resultados:**

1.355 individuos rechazaron la donación de piel. La tendencia de rechazos fue decreciente (2001-2009) en los grupos de edad de 0-11 años y de 12-19 años, y creciente en el grupo de ≥60 años. La tendencia siguió siendo decreciente (2010-2020) en el grupo de edad de 0-11 años y creciente en el de 20-40 años. El género masculino y los grupos de edad de 20-40 años, 41-59 años y ≥60 años presentaron, respectivamente, 27%, 34%, 47% y 53% menos probabilidades de rechazo.

**Conclusiones:**

es urgente la necesidad de medidas que busquen mitigar el alto número de rechazos asociados a la donación de piel.

## INTRODUCTION

Worldwide, more than 180,000 individuals die from burns each year, with the majority of these deaths occurring in lowand middle-income countries^(1)^. In Brazil, burns represent a significant public health issue. Annually, about 1,000,000 such accidents take place in the country, approximately half of which involve children^(2)^.

In more severe cases, the use of skin grafts for treating burns is essential. Skin grafting is an effective surgical strategy that involves transferring a segment of tissue from one part of the body to another to cover or repair a damaged area^(3)^. When an autograft is not possible, this treatment can be performed using the skin of a deceased donor^(3)^. Allografts can be harvested in cases of brain death or cardiopulmonary arrest of the donor^(4)^. The use of this graft can be life-sustaining for severely burned individuals^(3)^. Tissue from just a single donor can save up to 100 patients^(5)^.

In Brazil, allografts are used in burn patients with over 40% of their body surface affected and who lack viable options for temporary coverage of the injured area^(4)^. However, skin availability in the country is limited and difficult to obtain^(3)^. Demand still exceeds supply^(3)^. Only 42.7% of individuals who donated organs also consented to donate their skin^(6)^. Interestingly, in 2015, Australia had 84% more skin donors than Brazil, despite having a population size that is only 10% of Brazil’s^(7)^.

However, the refusal to donate skin is not a problem exclusive to the Brazilian system. In Iran, the average refusal rate was 51% during the period from 2007 to 2014^(5)^. In Mashhad, for example, the annual demand for skin allografts exceeding 3.5 million cm^2^ is not met by the available supply of about 20,000 cm^2(5)^. In the USA, between 30 and 50 active tissue banks have also reported difficulties^(8)^. Spain, which has the highest organ donation rates in the world, recorded about a 40% refusal rate for tissue donation^(9)^.

The reasons behind this phenomenon may be linked to family refusals to donate skin. In Brazil, the organ and tissue donation process is regulated by legislation that requires family authorization^(4)^. Technical issues with the donation, lack of team training, the manner in which the interview is conducted, the time required to complete the process, and the fear of body mutilation are factors associated with family refusals^(10)^.

Specific reasons for refusing skin donation are tied to a lack of awareness about the importance and feasibility of donating this tissue. The extraction of skin may evoke the notion of animalization of the donation, potentially leading to feelings of lost dignity for the deceased and negatively influencing family decision-making^(11)^. In addition to these factors, the low rate of skin donation linked to family refusal might also be related to other reasons, necessitating further investigations to explore various scenarios and the context of the COVID-19 pandemic.

Thus, the following question arises to be addressed by this study: “What are the trends and factors associated with the specific refusal of skin donation?”

## OBJECTIVES

To analyze the trends and factors associated with family refusal to donate skin for transplantation.

## METHODS

### Ethical Aspects

The study was conducted in accordance with national and international ethical guidelines and was approved by the Research Ethics Committee at the Hospital das Clínicas of the Faculty of Medicine, University of São Paulo. Consent was waived for the patients since the data are derived from individuals who had already passed away.

### Study Type

This is a quantitative, cross-sectional, analytical study with an exploratory and retrospective focus on specific refusals of skin donations from donors in brain death situations at an Organ Procurement Organization (OPO) in the State of São Paulo. The research followed the recommendations of the STROBE (Strengthening the Reporting of Observational Studies in Epidemiology) checklist.

### Data Source

The analytical material consisted of all copies of Organ and Tissue Authorization Terms signed by family members from January 2001 to December 2020. The study variables related to skin donation refusal include: year, donor age, gender, cause of death, type of hospital institution, and instances of skin donation refusals.

### Data Analysis

Data were collected and tabulated in Microsoft Excel® and analyzed using Stata 15.0 statistical software, both descriptively and inferentially. Temporal trend analysis was conducted through generalized linear regression using the Prais-Winsten method to calculate trends or percentage changes as indicated by the Annual Percent Change (APC) parameter. For interpreting the results, the following criteria were considered: an increasing trend - APC positive and p<0.05; a decreasing trend - APC negative and p<0.05; stationary, when the null hypothesis that there is no significant difference between the value of the variation and zero is accepted (p>0.05)^(12)^. Multivariable analysis was performed using multiple logistic regression to estimate the crude Odds Ratio (OR) and the respective 95% confidence intervals.

Based on these results, the final reduced model for the association between skin donation refusal and sociodemographic and corporate characteristics was estimated. Variables with a p-value of 0.25 or lower in the crude analyses were adopted. The stepwise method allowed the retention of variables with a p-value of 0.10 or lower in the regression analyses, and the final adjusted model included variables that maintained a p-value of less than 0.05. The fit of the final model was assessed using likelihood ratio statistics, the Wald test, and the coefficient of determination (R^2^). A significance level of 5% was maintained throughout the analysis.

## RESULTS

Among the data analyzed from 2,447 organ or tissue donors, 1,355 (55.37%) refused to donate skin. Gender and age group were associated with the refusal of skin donation (p=0.002 and p<0.001, respectively). The majority of individuals who refused skin extraction were male (55.94%) and belonged to the age group of 41 to 59 years (41.48%) (Table 1).

**Table 1:** Characterization of skin donations and refusals, considering the period from 2001 to 2020, São Paulo, São Paulo, Brazil, 2024

Variables	Refused n (%)	Donated n (%)	*p* value^‖^
Gender			0.002
Female	597 (44.06)	412 (37.73)	
Male	758 (55.94)	680 (62.27)	
Age Group			<0.001
Zero to 11 years	69 (5.09)	23 (2.10)	
12 to 19 years	128 (9.45)	75 (6.87)	
20 to 40 years	409 (30.18)	309 (28.30)	
41 to 59 years	562 (41.48)	496 (45.42)	
60 years and older	187 (13.80)	189 (17.31)	
Diagnosis			0.206
Cerebrovascular Accident	711 (52.47)	543 (49.73)	
Traumatic Brain Injury	391 (28.86)	364 (33.33)	
Anoxia	90 (6.64)	68 (6.23)	
External Causes	103 (7.60)	73 (6.68)	
Other^*^	60 (4.43)	44 (4.03)	
Type of Administration			0.091
Public Administration	733 (54.10)	628 (57.51)	
Private Administration	622 (45.90)	464 (42.49)	

‖ Chi-square test.

* Other conditions: cerebral abscess, cerebral edema, diffuse cerebral edema, diabetic ketoacidosis-related cerebral edema, cerebral edema due to exogenous intoxication, hepatic encephalopathy, viral encephalitis, hydrocephalus, arteriovenous malformation (AVM), meningoencephalitis, bacterial meningitis, meningococcal meningitis, pneumococcal meningitis, Dandy-Walker syndrome with hydrocephalus, brain tumor, pituitary tumor.

**Figure 1:**
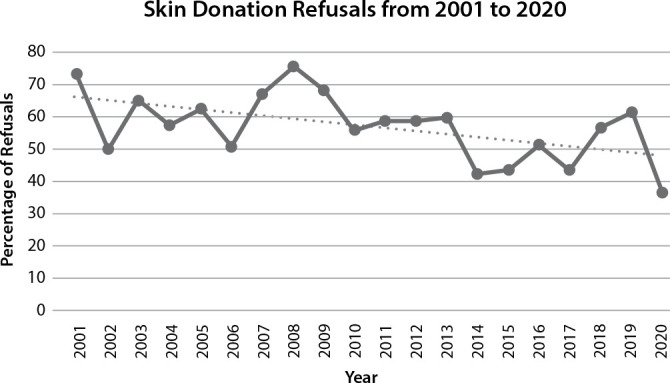
Temporal Evolution of Skin Donation Refusals from 2001 to 2020, São Paulo, São Paulo, Brazil, 2024

The year with the highest percentage of refusals was 2008; however, the years 2014 and 2020 saw a significant decrease in refusal rates (Figure 1).

Between 2001 and 2009, it is noticeable that the trend in the percentage of skin donation refusals decreased in the age groups of zero to 11 years (p<0.001) and 12 to 19 years (p=0.001). However, in the age group of 60 years or older, there was an increasing trend (p=0.016) during the same time period. From 2010 to 2020, the trend continued to decrease in the age group of zero to 11 years (p=0.012) and started to increase in the age group of 20 to 40 years (p<0.017) (Table 2).

**Table 2:** Temporal Trend of the Percentage of Skin Donation Refusals, by Characterization Variables, in Two Periods: 2001 to 2009 and 2010 to 2020, São Paulo, São Paulo, Brazil, 2024

Variables	2001 n (%)	2009 n (%)	APC^‡^ (IC95%)	∆%	*p* value	2010 n (%)	2020 n (%)	APC^‡^ (IC95%)	∆%	*p* value
Male	33 (75.00)	67 (56.78)	22.44 (-0.99; 56.54)	-24.29	0.316	49 (52.69)	32 (58.18)	-0.39 (-0.90; 2.63)	10.41	0.540
Public Administration	23 (52.27)	53 (44.92)	3.36 (-0.97; 65.96)	-14.06	0.509	51 (54.84)	35 (63.64)	9.47 (-0.51; 228.08)	16.04	0.116
Zero to 11 years	5 (11.36)	5 (4.24)	-0.87 (-0.94; 2.54)	-62.67	<0.001	3 (3.23)	0 (0)	-0.62 (-0.82; 0.28)	-100	0.012
12 to 19 years	9 (2.45)	6 (5.08)	-0.97 (-0.99; 7.70)	107.34	0.001	8 (8.60)	7 (12.73)	0.09 (-0.92; 14.48)	48.02	0.933
20 to 40 years	17 (38.64)	30 (25.42)	-0.82 (-0.99; 9.71)	-34.21	0.354	27 (29.03)	18 (32.73)	2.10 (0.28; 6.07)	12.74	0.017
41 to 59 years	11(25.00)	56 (47.46)	16.37 (-0.98; 707.93)	89.84	0.441	45 (48.39)	21 (38.18)	-0.06 (-0.79; 3.16)	-21.09	0.960
60 years and older	2 (4.55)	21 (17.80)	176.82 (2.54; 9119)	291.20	0.016	10 (10.75)	9 (16.36)	0.47 (-0.80; 10.74)	52.18	0.668
General population	73.33	68.21	13.79 (-0.90; 23.97)	-6.98	0.248	55.69	36.42	-0.90 (-0.99; 4.01)	-34.60	0.215

*‡Annual Percent Change, Calculated Based on β1 from the Prais-Winsten Regression.*

Considering the gender variable, from 2001 to 2020, male potential donors had a 24% lower likelihood of having their skin donation refused compared to females (p=0.002). Between 2010 and 2020, males had a 31% lower chance of refusal for skin extraction (p<0.001)

From 2001 to 2020, the age group of 12 to 19 years experienced a 44% lower chance of skin donation refusal (p=0.045), the 20 to 40 years group had a 56% lower chance (p=0.001), individuals aged 41 to 59 years showed a 63% lower chance (p<0.001), and those aged 60 years or older had a 68% lower chance (p<0.001), when compared to those aged zero to 11 years.

Between 2001 and 2009, individuals aged 12 to 19 years had an 81% lower likelihood of skin donation refusal (p=0.006), those aged 20 to 40 years had 79% lower chances (p=0.004), those aged 41 to 59 years had 83% lower chances (p=0.001), and those aged 60 years or older had 86% lower chances (p=0.001). It is noteworthy that, from 2010 to 2020, individuals aged 60 years or older had a 47% lower chance of having their skin donation refused (p=0.045) (Table 3).

**Table 3:** Association Between Refusal of Skin Donation and Sociodemographic and Clinical Characteristics, Considering Crude *Odds Ratios*, São Paulo, São Paulo, Brazil, 2024

Variables	2001 - 2009		2010 - 2020		2001 - 2020	
	OR (95% CI)	*p* value	OR (95% CI)	*p* value	OR (95% CI)	*p* value
Gender						
Female	1	-	1	-	1	-
Male	1.05 (0.78; 1.43)	0.712	0.69 (0.56; 0.84)	<0.001	0.76 (0.65; 0.90)	0.002
Age Group						
Zero to 11 years	1	-	1	-	1	-
12 to 19 years	0.19 (0.06; 0.62)	0.006	0.98 (0.50; 1.90)	0.957	0.56 (0.32; 0.98)	0.045
20 to 40 years	0.21 (0.07; 0.61)	0.004	0.66 (0.36; 1.20)	0.182	0.44 (0.26; 0.72)	0.001
41 to 59 years	0.17 (0.05; 0.49)	0.001	0.58 (0.32; 1.05)	0.075	0.37 (0.23; 0.61)	<0.001
60 years and older	0.14 (0.04; 0.42)	0.001	0.53 (0.28; 0.98)	0.045	0.32 (0.19; 0.55)	<0.001
Diagnosis						
Cerebrovascular Accident	0.81 (0.37; 1.78)	0.617	1.01 (0.62; 1.63)	0.956	0.96 (0.64; 1.43)	0.844
Traumatic Brain Injury	0.88 (0.38; 2.02)	0.769	0.83 (0.51; 1.35)	0.462	0.78 (0.52; 1.19)	0.259
Anoxia	1.38 (0.48; 3.91)	0.543	0.91 (0.51; 1.64)	0.771	0.97 (0.58; 1.60)	0.907
External Causes	0.76 (0.33; 1.74)	0.523	0.78 (0.35; 1.72)	0.545	1.03 (0.63; 1.69)	0.892
Other	1		1		1	-
Type of Administration						
Public Administration	0.97 (0.72; 1.32)	0.879	0.92 (0.75; 1.11)	0.407	0.87 (0.74; 1.02)	0.091
Private Administration	1		1		1	-

*OR - Odds Ratio.*

In the final reduced model for the period from 2010 to 2020, male individuals had a 34% lower likelihood of having their skin donation refused compared to females (OR = 0.66; CI: 0.54-0.80; p<0.001). Throughout the entire period from 2001 to 2020, this likelihood was 27% lower for males (OR = 0.73; CI: 0.61-0.86; p<0.001).

Considering age as a variable in the final reduced model, from 2001 to 2009, individuals aged 12 to 19 years had an 81% lower chance of having their skin donation refused compared to those aged zero to 11 years (OR = 0.19; CI: 0.06-0.62; p=0.006). For the age group of 20 to 40 years, the reduction was 79% (OR = 0.21; CI: 0.07-0.61; p=0.004), and for those aged 41 to 59 years, it was 83% (OR = 0.17; CI: 0.05-0.49; p=0.001), while for individuals aged 60 years or older, it was 86% (OR = 0.14; CI: 0.04-0.42; p=0.001).

From 2010 to 2020, the age group of 41 to 59 years had an OR of 0.75 (CI: 0.61-0.92; p=0.008) and the age group of 60 years or older had an OR of 0.67 (CI: 0.51-0.89; p=0.007), indicating that donors in these age groups had, respectively, 25% and 33% lower chances of having their skin donation refused. For the age group of 20 to 40 years, from 2001 to 2020, the reduction was 34% (OR = 0.66; CI: 0.49-0.87; p=0.004). In the age group of 41 to 59 years, it was 47% (OR = 0.53; CI: 0.41-0.70; p<0.001) and for those 60 years or older, it was 53% (OR = 0.47; CI: 0.34-0.64; p<0.001), over the same time frame (Table 4).

**Table 4:** Final Reduced Model of Association Between Skin Donation Refusal and Sociodemographic Characteristics, São Paulo, São Paulo, Brazil, 2024

Variables	2001 - 2009^§^		2010 - 2020^§§^		2001 - 2020^§§§^	
	OR (95% CI)	*p* value	OR (95% CI)	*p* value	OR (95% CI)	*p* value
Gender						
Male	-	-	0.66 (0.54; 0.80)	<0.001	0.73 (0.61; 0.86)	<0.001
Age Group						
12 to 19 years	0.19 (0.06; 0.62)	0.006	-	-	-	-
20 to 40 years	0.21 (0.07; 0.61)	0.004	-	-	0.66 (0.49; 0.87)	0.004
41 to 59 years	0.17 (0.05; 0.49)	0.001	0.75 (0.61; 0.92)	0.008	0.53 (0.41; 0.70)	<0.001
60 years and older	0.14 (0.04; 0.42)	0.001	0.67 (0.51; 0.89)	0.007	0.47 (0.34; 0.64)	<0.001

§ R^2^: 1.94%; p=0.001;

§§ R^2^: 1.02%; p<0.001;

§§§ R^2^: 1.12%; p<0.001; OR - Odds Ratio

## DISCUSSION

Our results highlight a high rate of specific family refusals to donate skin. This may be related to several factors that contribute to the lower social impact of tissues used for donations compared to other organs^(13)^.

Generally, the lack of social awareness about skin donation, concerns about body image, misinformation, and in some cases, the inadequate or nonexistent approach by healthcare professionals during family interviews are the main factors influencing and contributing to the high number of refusals observed^(11)^.

A study by Brazilian researchers found that a lack of understanding of brain death, religious beliefs, insufficient time for decision-making, and the lack of technical competence of hospital staff also influence family refusals^(14)^. Furthermore, research in Spain has shown that despite a strong tradition of organ transplantation in various health institutions, skin donation is largely an unknown process and is only part of routine activities at a few health centers. Both situations corroborate and justify the high rates of tissue refusals^(15)^.

The findings of this study also revealed that the age and gender of the donor are factors that weaken the skin donation process and contribute to the increase in family refusals. Although descriptive data from the investigation revealed that males have a higher number of refusals, the logistic regression model demonstrated that women are more likely to have their skin extraction refused.

Males, compared to females, also showed lower chances of refusal in a study conducted in Saudi Arabia^(16)^, indicating that this scenario is not exclusively a national problem. It is assumed that this is linked to the stereotyped image of female skin as a representation of care and delicacy. Therefore, the acceptance for donation from male individuals is higher, considering its social character, which could be an important determinant for family consent^(17)^. It is worth noting that this phenomenon may be a consequence of historically and socially imposed beauty standards, where female skin is seen as a symbol of perfection, an ideal to be venerated and preserved, something that should not be extracted or supposedly violated in the event of a donation^(18)^.

The investigation also revealed that the older the donor, the greater the likelihood of family refusal to donate skin. Skin is considered a societal calling card; thus, there is significant concern for its preservation^(19)^. Consequently, donating skin can symbolically represent an invasion of the donor’s privacy and dignity, since touching is viewed as an act of intimacy, a privilege, and a transcendence of social barriers^(18)^. Prejudice against older skin is also a notable factor in the process^(19)^. Age can influence family decisions and also affect the offering of tissue by interviewers in cases involving older individuals. Analysis of the profile of skin donors at a Brazilian center shows that the maximum age for skin donation was 60 years, with an average age of 41 years^(20)^.

It is evident that changes in public policies initiated in 2009 might explain the reduction in refusals from that period. In that year, the Brazilian transplant system underwent restructuring through legislation^(4)^, which may have optimized skin donation.

It is important to note that the decrease in the refusal rate for skin donation in 2014 might be linked to the fire at the Kiss nightclub in Santa Maria - RS, Brazil, in 2013. This national tragedy may have sensitized the population to skin donation, highlighting a critical deficit of grafts needed for treating the victims in Brazilian skin banks.

It is assumed that the data presented in 2020 also reflect a widespread awareness campaign about organ and tissue donation conducted by the family of a renowned Brazilian artist who died in November 2019. Following the event, data from Google Trends recorded an increase in searches for “Organ and Tissue Donation” and “Brain Death”^(21)^.

It is concerning to observe that the trend of refusal remained stationary during some periods. This highlights the impact of failing to adopt or adhere to effective measures to alter the situation, emphasizing the need for the development and dissemination of educational initiatives and campaigns focused on skin donation, as well as the training of professionals in the field^(6)^.

### Study limitations

This study has considerable limitations. Although the analytical models were significant, it can be concluded that the variables studied are insufficient to fully explain the outcomes of skin donation refusal. Further scenarios should be considered, such as analyzing data from other regions of Brazil and investigating the characteristics or motivations of the family members of skin donors.

### Contributions to the Field of Nursing

Our findings underscore the importance of skin donation and the need for more in-depth discussions on the subject, emphasizing the crucial role played by nursing in this process. In family interviews, these professionals have an advantage in influencing positive outcomes by clarifying doubts and alleviating fears, which facilitates the decision-making process for donations. Additionally, nurses have the capability to propose and support public policies aimed at reducing the high rate of refusals. Our study also identified a significant research gap in the field, highlighting the need for more studies that examine both the obstacles and strategies to improve the donation process. Moreover, we emphasize the importance of developing and implementing evidence-based intervention proposals that can be integrated into training and practice for nurses, strengthening their roles in advocacy and public health education.

## CONCLUSIONS

There is a clear need for measures aimed at mitigating the high number of family refusals associated with skin donation. In this context, identifying specific characteristics of refusals may provide a solution to this issue. As the current analysis has shown, it is evident that female donors and those in older age groups tend to have their tissue refused, varying according to the period analyzed. These data suggest that other factors may be associated with family refusal of skin donation, such as lack of awareness, prejudices, and low visibility around the issue, which impact family decision-making. Therefore, it is imperative to deepen research in this area and verify data like those from the current study in order to support actions and strategies to increase the number of skin donations and reduce refusals. Social barriers surrounding the topic can be mitigated with educational measures linked to universities and major media outlets.
