# Supplementary material for: Family refusal of skin donation for transplantation: trends and associated factors
Source: Rev Bras Enferm. 2024 Jul 29;77(3):e20230209. doi: 10.1590/0034-7167-2023-0209 (PMC11290726; doi:10.1590/0034-7167-2023-0209)
Supplement: Translation

# Translation: Recusa familiar de doação de pele para transplante: tendência e fatores associados

**DOI:** 10.1590/0034-7167-2023-0209pt

**Authors:** Karoline de Oliveira Lins Souto, Rafael Rodrigo da Silva Pimentel, Ágata Nunes Brito, Edvaldo Leal de Moraes, Marcelo José dos Santos

**Affiliations:** I Universidade de São Paulo. São Paulo, São Paulo, Brasil; II Universidade de São Paulo, Hospital das Clínicas, Organização de Procura de Órgãos. São Paulo, São Paulo, Brasil

**Keywords:** Doação de Pele, Banco de Tecidos, Recusa Familiar, Enfermagem em Saúde Pública, Enfermagem

## Abstract

**Objetivos:**

analisar a tendência e os fatores associados à recusa familiar de doação de pele para transplante.

**Métodos:**

estudo transversal desenvolvido no Estado de São Paulo com os termos de autorização familiar firmados entre 2001 e 2020. As variáveis analisadas foram: ano, idade, sexo, causa do óbito e tipo da instituição. Os dados foram analisados por meio de regressão linear e logística múltipla, com o *Odds Ratio* estimado, adotando-se p<0,05 como significância estatística.

**Resultados:**

1.355 indivíduos recusaram a doação de pele. A tendência de recusas foi decrescente (2001-2009) nas faixas etárias de 0-11 anos e de 12-19 anos e crescente na faixa de ≥60 anos. A tendência permaneceu decrescente (2010-2020) na faixa etária de 0-11 anos e crescente na de 20-40 anos. O sexo masculino e as faixas etárias de 20-40 anos, 41-59 anos e ≥60 anos apresentaram, respectivamente, 27%, 34%, 47% e 53% menores chances de recusa.

**Conclusões:**

é urgente a necessidade de medidas que visem atenuar o alto número de recusas associadas à doação de pele.

## INTRODUÇÃO

No mundo, mais de 180.000 indivíduos morrem de queimaduras a cada ano, e a maioria desses óbitos ocorre em países de baixa e média renda^(1)^. No Brasil, as queimaduras representam um problema significativo para a saúde pública. Anualmente, no país, ocorrem cerca de 1.000.000 de acidentes desse tipo; dos quais, aproximadamente metade das ocorrências acontece com crianças^(2)^.

Em casos mais graves, o uso de enxerto de pele para o tratamento das queimaduras torna-se fundamental. O enxerto de pele é uma estratégia eficaz e cirúrgica que envolve a retirada de um segmento de tecido de uma parte do corpo para outra, visando cobrir ou reparar uma área danificada^(3)^. Na impossibilidade de um autoenxerto, esse tratamento pode ser feito com a pele de um doador falecido^(3)^. O aloenxerto pode ser extraído em casos de morte encefálica ou parada cardiorrespiratória do doador^(4)^. O uso desse enxerto pode significar a continuidade da vida para os gravemente queimados^(3)^. O tecido de apenas um único doador pode salvar a vida de até 100 pacientes^(5)^.

No Brasil, o aloenxerto é usado em pacientes queimados com mais de 40% da superfície corporal atingida e que não possuem viabilidade para cobertura temporária da área lesionada^(4)^. Contudo, no país, a disponibilidade de pele é limitada e de difícil obtenção^(3)^. A demanda ainda é superior à oferta^(3)^. No país, somente 42,7% dos indivíduos que doaram órgãos também aceitaram a doação da pele^(6)^. É interessante observar que, em 2015, a Austrália teve 84% a mais de doadores de pele do que o Brasil, com um número de habitantes equivalente a 10% da população brasileira^(7)^.

Entretanto, a recusa de doação de pele não é um problema exclusivo do sistema brasileiro. No Irã, a taxa média de recusa foi de 51% no período de 2007-2014^(5)^. Em Mashhad, por exemplo, a necessidade de aloenxerto de pele de mais de 3,5 milhões de cm^2^, anualmente, não é suprida pela oferta de cerca de 20.000 cm^2(5)^. Nos EUA, entre 30 e 50 bancos de tecidos ativos também relataram dificuldades^(8)^. A Espanha possui as mais altas taxas de doação de órgãos do mundo, mas registrou cerca de 40% de recusa de doação de tecidos^(9)^.

As causas que explicam esse fenômeno podem estar ligadas à recusa familiar para a doação de pele. No Brasil, o processo de doação de órgãos e tecidos é regulamentado por legislação que prevê a autorização familiar para a sua realização^(4)^. As questões técnicas da doação, a falta de capacitação da equipe, a forma de realizar a entrevista, o tempo necessário para conclusão do processo e o medo de mutilação do corpo são fatores associados à recusa familiar^(10)^.

Os motivos específicos para a recusa da doação de pele estão vinculados ao desconhecimento da importância e possibilidade da doação deste tecido, sendo que a extração da pele pode trazer a ideia de animalização da doação, o que pode gerar o sentimento de perda da dignidade da pessoa falecida e influenciar negativamente na tomada de decisão familiar^(11)^. Além desses fatores, a baixa doação de pele ligada à recusa familiar também pode estar associada a outros motivos, fazendo-se necessário a realização de novas investigações que explorem os diversos cenários e o contexto da pandemia da covid-19.

Com isso, emerge o seguinte questionamento a ser respondido por esse estudo: “Quais são as tendências e os fatores associados à recusa específica de doação de pele?”.

## OBJETIVO

Analisar a tendência e os fatores associados a recusa familiar de doação de pele para transplante.

## MÉTODOS

### Aspectos éticos

O estudo foi conduzido de acordo com as diretrizes de ética nacionais e internacionais e foi aprovado pelo Comitê de Ética em Pesquisa do Hospital das Clínicas da Faculdade de Medicina da Universidade de São Paulo. O consentimento dos pacientes foi dispensado, uma vez que os dados são secundários de indivíduos que já vieram a óbito.

### Tipo do estudo

Trata-se de um estudo quantitativo do tipo transversal analítico, de caráter exploratório e retrospectivo, sobre as recusas específicas de pele de doadores em situação de morte encefálica de uma Organização de Procura de Órgãos (OPO) do Estado de São Paulo. A pesquisa seguiu as recomendações do *checklist STrengthening the Reporting of OBservational studies in Epidemiology* (STROBE).

### Fonte de dados

O material de análise foi composto por todas as cópias dos Termos de Autorização de Órgãos e Tecidos firmados por familiares entre janeiro de 2001 e dezembro de 2020. As variáveis do estudo relacionadas à recusa de pele são: ano, idade do doador, sexo, causa do óbito, tipo da instituição hospitalar e recusas de doações de pele.

### Análise dos dados

Os dados foram coletados e tabulados em planilha de *Microsoft Excel* ^®^ e analisados no software de estatística Stata 15.0, de forma descritiva e inferencial, com a análise de tendência temporal por meio de regressão linear generalizada do tipo Prais-Winsten para calcular a tendência ou mudança percentual evidenciada pelo parâmetro *Annual Percent Change* (APC). Para a interpretação dos resultados foi considerado: tendência crescente - APC positiva e p<0,05; decrescente - APC negativa e p<0,05; estacionária, quando se aceita a hipótese nula de que não há diferença significativa entre o valor da variação e zero (p>0,05)^(12)^. Foi realizada análise multivariável do tipo regressão logística múltipla, estimando-se o *Odds Ratio* (OR) bruto e respectivos intervalos de confiança a 95%.

Com base nestes resultados, estimou-se o modelo final reduzido de associação entre recusa de doação de pele e características sociodemográficas e de razão social. Adotando-se as variáveis com valor de p igual ou inferior a 0,25 nas análises brutas. O método *stepwise* permitiu manter as variáveis com valor de p igual ou inferior a 0,10 nas análises de regressão e o modelo final ajustado foi formado com as variáveis que se mantiveram com valor de p inferior a 0,05. A avaliação do ajuste do modelo final deu-se por meio da estatística de razão de verossimilhança, do teste de Wald e do coeficiente de determinação (R^2^). O nível de significância adotado em toda a análise foi de 5%.

## RESULTADOS

Dentre os dados analisados de 2.447 doadores de algum órgão ou tecido, 1.355 (55,37%) recusaram a doação de pele. O sexo e a faixa etária foram associados à recusa de pele (p=0,002 e p<0,001, respectivamente). A maioria dos indivíduos que recusou a extração de pele era do sexo masculino (55,94%) e pertencia à faixa etária de 41 a 59 anos (41,48%) (Tabela 1).

**Tabela 1:** Caracterização de doações e recusas de pele, considerando o intervalo 2001 a 2020, São Paulo, São Paulo, Brasil, 2024

Variáveis	Recusado n (%)	Doado n (%)	Valor de *p* ^‖^
Sexo			0,002
Feminino	597 (44,06)	412 (37,73)	
Masculino	758 (55,94)	680 (62,27)	
Faixa etária			<0,001
Zero a 11 anos	69 (5,09)	23 (2,10)	
12 a 19 anos	128 (9,45)	75 (6,87)	
20 a 40 anos	409 (30,18)	309 (28,30)	
41 a 59 anos	562 (41,48)	496 (45,42)	
Maior ou igual a 60 anos	187 (13,80)	189 (17,31)	
Diagnóstico			0,206
Acidente Vascular Encefálico	711 (52,47)	543 (49,73)	
Traumatismo Crânio Encefálico	391 (28,86)	364 (33,33)	
Anoxia	90 (6,64)	68 (6,23)	
Causas externas	103 (7,60)	73 (6,68)	
Outros^*^	60 (4,43)	44 (4,03)	
Razão social			0,091
Administração pública	733 (54,10)	628 (57,51)	
Administração privada	622 (45,90)	464 (42,49)	

‖ Teste qui-quadrado.

* Outros: Abcesso cerebral, edema cerebral, edema cerebral difuso, edema cerebral por cetoacidose diabética, edema cerebral por intoxicação exógena, encefalopatia hepática, encefalite viral, hidrocefalia, MAV (malformação arteriovenosa), meningoencefalite, meningite bacteriana, meningite meningocócica, meningite pneumocócica, sìndrome de Deandy-Walker+Hidrocefalia, tumor cerebral, tumor de hipófise.

O ano com o maior percentual de recusas foi 2008, em contrapartida, os anos de 2014 e 2020 registraram queda expressiva nas taxas de recusas (Figura 1).

**Figura 1:** Evolução temporal das recusas de doação de pele, entre 2001 e 2020, São Paulo, São Paulo, Brasil, 2024

No período de 2001 a 2009, é possível perceber que a tendência temporal do percentual de recusas de doação de pele foi decrescente nas faixas etárias de zero a 11 anos (p<0,001) e de 12 a 19 anos (p=0,001). Já na faixa etária de 60 anos ou mais, observou-se uma tendência crescente (p=0,016) nesse mesmo intervalo de tempo. No período de 2010 a 2020, a tendência permaneceu decrescente na faixa de idade de zero a 11 anos (p=0,012) e tornou-se crescente na faixa etária de 20 a 40 anos (p<0,017) (Tabela 2).

**Tabela 2:** Tendência temporal do percentual de recusas de doação de pele, por variáveis de caracterização, em dois períodos: de 2001 a 2009 e 2010 a 2020, São Paulo, São Paulo, Brasil, 2024

Variáveis	2001 n (%)	2009 n (%)	APC^‡^ (IC95%)	∆%	Valor de *p*	2010 n (%)	2020 n (%)	APC^‡^ (IC95%)	∆%	Valor de *p*
Sexo masculino	33 (75,00)	67 (56,78)	22,44 (-0,99; 56,54)	-24,29	0,316	49 (52,69)	32 (58,18)	-0,39 (-0,90; 2,63)	10,41	0,540
Setor público	23 (52,27)	53 (44,92)	3,36 (-0,97; 65,96)	-14,06	0,509	51 (54,84)	35 (63,64)	9,47 (-0,51; 228,08)	16,04	0,116
Zero a 11 anos	5 (11,36)	5 (4,24)	-0,87 (-0,94; 2,54)	-62,67	<0,001	3 (3,23)	0 (0)	-0,62 (-0,82; 0,28)	-100	0,012
12 a 19 anos	9 (2,45)	6 (5,08)	-0,97 (-0,99; 7,70)	107,34	0,001	8 (8,60)	7 (12,73)	0,09 (-0,92; 14,48)	48,02	0,933
20 a 40 anos	17 (38,64)	30 (25,42)	-0,82 (-0,99; 9,71)	-34,21	0,354	27 (29,03)	18 (32,73)	2,10 (0,28; 6,07)	12,74	0,017
41 a 59 anos	11(25,00)	56 (47,46)	16,37 (-0,98; 707,93)	89,84	0,441	45 (48,39)	21 (38,18)	-0,06 (-0,79; 3,16)	-21,09	0,960
Maior ou igual a 60 anos	2 (4,55)	21 (17,80)	176,82 (2,54; 9119)	291,20	0,016	10 (10,75)	9 (16,36)	0,47 (-0,80; 10,74)	52,18	0,668
População geral	73,33	68,21	13,79 (-0,90; 23,97)	-6,98	0,248	55,69	36,42	-0,90 (-0,99; 4,01)	-34,60	0,215

*‡Annual Percent Change, calculada com base no β1 da regressão de Prais-Winsten.*

Considerando a variável sexo, no período de 2001 a 2020, os potenciais doadores do sexo masculino tiveram 24% menor chance de terem sua extração de pele recusada quando comparados ao sexo feminino (p=0,002). Já entre 2010 e 2020, os indivíduos do sexo masculino apresentaram 31% menor chance de recusa para a extração da pele (p<0,001).

No período de 2001 a 2020, a faixa etária de 12 a 19 anos teve 44% menor chance de ter sua extração de pele recusada (p=0,045), a de 20 a 40 anos teve 56% menor chance (p=0,001), 41 a 59 anos apresentou 63% menor chance (p<0,001) e pessoas com 60 anos ou mais tiveram 68% menor chance (p<0,001), quando comparadas às de zero a 11 anos.

Entre 2001 e 2009, os indivíduos de 12 a 19 anos tiveram 81% menores chances de ter sua extração de pele recusada (p=0,006), de 20 a 40 anos tiveram 79% menores chances (p=0,004), de 41 a 59 anos tiveram 83% menores chances (p=0,001) e de 60 anos ou mais, 86% menores chances (p=0,001). É importante destacar que, no período de 2010 a 2020, os indivíduos com 60 anos ou mais apresentaram 47% menor chance de ter sua doação de pele recusada (p=0,045) (Tabela 3).

**Tabela 3:** Associação entre recusa da doação de pele e características sociodemográficas e clínicas, considerando-se *Odds Ratio* bruto, São Paulo, São Paulo, Brasil, 2024

Variáveis	2001 a 2009		2010 a 2020		2001 a 2020	
	OR (IC95%)	Valor de *p*	OR (IC95%)	Valor de *p*	OR (IC95%)	Valor de *p*
Sexo						
Feminino	1	-	1	-	1	-
Masculino	1,05 (0,78; 1,43)	0,712	0,69 (0,56; 0,84)	<0,001	0,76 (0,65; 0,90)	0,002
Faixa etária						
Zero a 11 anos	1	-	1	-	1	-
12 a 19 anos	0,19 (0,06; 0,62)	0,006	0,98 (0,50; 1,90)	0,957	0,56 (0,32; 0,98)	0,045
20 a 40 anos	0,21 (0,07; 0,61)	0,004	0,66 (0,36; 1,20)	0,182	0,44 (0,26; 0,72)	0,001
41 a 59 anos	0,17 (0,05; 0,49)	0,001	0,58 (0,32; 1,05)	0,075	0,37 (0,23; 0,61)	<0,001
Maior ou igual a 60 anos	0,14 (0,04; 0,42)	0,001	0,53 (0,28; 0,98)	0,045	0,32 (0,19; 0,55)	<0,001
Diagnóstico						
Acidente Vascular Encefálico	0,81 (0,37; 1,78)	0,617	1,01 (0,62; 1,63)	0,956	0,96 (0,64; 1,43)	0,844
Traumatismo Crânio Encefálico	0,88 (0,38; 2,02)	0,769	0,83 (0,51; 1,35)	0,462	0,78 (0,52; 1,19)	0,259
Anoxia	1,38 (0,48; 3,91)	0,543	0,91 (0,51; 1,64)	0,771	0,97 (0,58; 1,60)	0,907
Causas externas	0,76 (0,33; 1,74)	0,523	0,78 (0,35; 1,72)	0,545	1,03 (0,63; 1,69)	0,892
Outros	1		1		1	-
Razão social						
Administração pública	0,97 (0,72; 1,32)	0,879	0,92 (0,75; 1,11)	0,407	0,87 (0,74; 1,02)	0,091
Administração privada	1		1		1	-

*OR - Odds Ratio.*

No modelo final reduzido, no período de 2010 a 2020, os indivíduos do sexo masculino apresentaram 34% menores chances de terem sua doação de pele recusada em comparação com pessoas do sexo feminino (OR = 0,66; IC: 0,54-0,80; p<0,001). Em todo o período de 2001 a 2020, essa chance foi 27% menor para os indivíduos do sexo masculino (OR = 0,73; IC: 0,61-0,86; p<0,001).

Considerando a faixa etária como variável no modelo final reduzido, de 2001 a 2009, indivíduos de 12 a 19 anos tiveram 81% menor chance de ter sua doação de pele recusada em comparação com aqueles de zero a 11 anos (OR = 0,19; IC: 0,06-0,62; p=0,006). Para a faixa de 20 a 40 anos, a redução foi de 79% (OR = 0,21; IC: 0,07-0,61; p=0,004) e para a faixa de 41 a 59 anos, de 83% (OR = 0,17; IC: 0,05-0,49; p=0,001), enquanto que para indivíduos de 60 anos ou mais, foi de 86% (OR = 0,14; IC: 0,04-0,42; p=0,001).

No período de 2010 a 2020, a faixa etária de 41 a 59 anos teve um OR de 0,75 (IC: 0,61-0,92; p=0,008) e a faixa de 60 anos ou mais, um OR de 0,67 (IC: 0,51-0,89; p=0,007), indicando que doadores dessas idades tiveram, respectivamente, 25% e 33% menos chances de ter sua doação de pele recusada. Para a faixa de 20 a 40 anos, de 2001 a 2020, a redução foi de 34% (OR = 0,66; IC: 0,49-0,87; p=0,004). Na faixa de 41 a 59 anos, foi de 47% (OR = 0,53; IC: 0,41-0,70; p<0,001) e para 60 anos ou mais, de 53% (OR = 0,47; IC: 0,34-0,64; p<0,001), no mesmo intervalo de tempo (Tabela 4).

**Tabela 4:** Modelo final reduzido de associação entre recusa da doação de pele e características sociodemográficas, São Paulo, São Paulo, Brasil, 2024

Variáveis	2001 a 2009^§^		2010 a 2020^§§^		2001 a 2020^§§§^	
	OR (IC95%)	Valor de *p*	OR (IC95%)	Valor de *p*	OR (IC95%)	Valor de *p*
Sexo						
Masculino	-	-	0,66 (0,54; 0,80)	<0,001	0,73 (0,61; 0,86)	<0,001
Faixa etária						
12 a 19 anos	0,19 (0,06; 0,62)	0,006	-	-	-	-
20 a 40 anos	0,21 (0,07; 0,61)	0,004	-	-	0,66 (0,49; 0,87)	0,004
41 a 59 anos	0,17 (0,05; 0,49)	0,001	0,75 (0,61; 0,92)	0,008	0,53 (0,41; 0,70)	<0,001
Maior ou igual a 60 anos	0,14 (0,04; 0,42)	0,001	0,67 (0,51; 0,89)	0,007	0,47 (0,34; 0,64)	<0,001

§ R^2^: 1,94%; p=0,001;

§§ R^2^: 1,02%; p<0,001;

§§§ R^2^: 1,12%; p<0,001; OR - Odds Ratio.

## DISCUSSÃO

Nossos resultados evidenciam alta recusa familiar específica de doação de pele. Esse fato pode estar relacionado a uma série de fatores que contribuem para uma menor repercussão social dos tecidos que podem ser utilizados em doações, em comparação a outros órgãos^(13)^. De forma geral, o desconhecimento social sobre a doação de pele, a preocupação em relação à imagem corporal, a falta de informação e uma abordagem inadequada e, em alguns casos, inexistente dos profissionais de saúde durante a entrevista familiar são os principais fatores que influenciam e contribuem com o alto número de recusas encontrado^(11)^.

Um estudo de pesquisadores brasileiros identificou que também influenciam na recusa familiar a não compreensão do conceito de morte encefálica, crenças religiosas, a insuficiência no tempo para tomada de decisão e a falta de competência técnica da equipe hospitalar^(14)^. Ademais, uma pesquisa da Espanha constatou que, apesar da grande tradição em transplante de órgãos em variadas instituições de saúde, a doação de pele é um processo em grande parte desconhecido e faz parte da rotina de atividades regulares de poucos centros de saúde. Ambas as situações corroboram e justificam as altas taxas de recusa de tecidos^(15)^.

Os achados do presente estudo ainda evidenciaram que a idade e o sexo do doador são fatores que fragilizam o processo de doação de pele e corroboram com o aumento da recusa familiar. Embora os dados descritivos da investigação tenham revelado que o sexo masculino tem um maior número de recusas, o modelo de regressão logística demonstrou que as mulheres possuem uma maior chance de terem sua extração de pele recusada.

A pessoa do sexo masculino, quando comparada ao sexo feminino, também apresentou menores chances de recusa em uma pesquisa realizada na Arábia Saudita^(16)^, demonstrando que tal cenário não é exclusivamente uma problemática nacional. Supõe-se que tal fato esteja ligado à imagem estereotipada da pele feminina como representação de cuidado e delicadeza. Sendo assim, a aceitação para doação do indivíduo do sexo masculino é maior, tendo em vista seu caráter social, podendo configurar como um importante determinante para o consentimento familiar^(17)^. É válido ressaltar que esse fenômeno pode ser consequência dos padrões de beleza impostos, histórica e socialmente, nos quais a pele feminina seria um símbolo de perfeição, um ideário a ser cultuado e preservado, algo que não pudesse ser extraído ou supostamente violado no caso de uma doação^(18)^.

A investigação ainda revelou que quanto maior a idade do doador, maior é a chance de recusa familiar de doação de pele. A pele é considerada o cartão de visita para a sociedade; portanto, existe uma grande preocupação com a sua preservação^(19)^. Dessa forma, a doação dessa estrutura pode simbolizar socialmente uma invasão da privacidade e da dignidade do doador, pois tocar é visto como um ato de intimidade, um privilégio, uma transcendência de barreiras sociais^(18)^. O preconceito em torno da pele idosa também é uma variante considerável no processo^(19)^. A idade pode influenciar na decisão da família e também na oferta do tecido pelos entrevistadores nos casos de indivíduos com idade elevada. Ao analisar o perfil de doadores de pele de um centro brasileiro, nota-se que a idade máxima de doação de pele foi de 60 anos e a média de 41 anos^(20)^.

Percebe-se que as alterações nas políticas públicas iniciadas em 2009 podem ser a explicação para a redução das recusas a partir desse período. Nesse ano, o sistema brasileiro de transplantes foi reestruturado por meio da legislação^(4)^, o que pode ter otimizado a doação de pele.

É válido ressaltar que a redução da taxa de recusa de doação de pele, no ano de 2014, pode estar ligada ao incêndio ocorrido na boate Kiss, localizada em Santa Maria - RS, Brasil, em 2013. A tragédia de comoção nacional pode ter sensibilizado a população para a doação de pele, após evidenciar um déficit de enxertos, extremamente necessário ao tratamento das vítimas, nos bancos de pele brasileiros.

Supõe-se que os dados apresentados em 2020 também sejam reflexo de uma campanha de ampla divulgação sobre a doação de órgãos e tecidos realizada pela família de um famoso artista brasileiro falecido em novembro de 2019. Após o fato, dados obtidos pelo *Google Trends* registraram um aumento no número de pesquisas com as expressões “Doação de Órgãos e Tecidos” e “Morte Encefálica”^(21)^.

É preocupante verificar que a tendência de recusa se manteve estacionária em alguns períodos. Esse dado reforça o impacto da não adoção ou a adesão a medidas ineficazes para alteração da situação, o que explicita a necessidade da elaboração e divulgação de ações educativas e campanhas voltadas à doação de pele, além da qualificação de profissionais da área^(6)^.

### Limitações do estudo

Esse estudo apresenta limitações a serem consideradas. Embora os modelos de análise tenham sido significativos, pode-se concluir que as variáveis estudadas são insuficientes para explicar o desfecho de recusa de doação de pele. Existem outros cenários a serem considerados, como a análise de dados de outras regiões do Brasil e a investigação de características ou motivações dos familiares dos doadores de pele.

### Contribuições para a Área da Enfermagem

Nossos achados destacam a importância da doação de pele e a necessidade de discussões mais aprofundadas sobre o tema, enfatizando o papel crucial desempenhado pela Enfermagem neste processo. Em entrevistas familiares, esses profissionais têm uma vantagem para influenciar desfechos positivos, ao esclarecer dúvidas e mitigar receios, o que facilita a decisão pela doação. Além disso, os enfermeiros têm a capacidade de propor e apoiar políticas públicas voltadas à redução do alto índice de recusas. Nosso estudo também identificou uma significativa lacuna de pesquisas na área, ressaltando a necessidade de mais estudos que examinem tanto os obstáculos quanto as estratégias para melhorar o processo de doação. Adicionalmente, enfatizamos a importância de desenvolver e implementar propostas de intervenção baseadas em evidências, que possam ser integradas ao treinamento e à prática dos enfermeiros, fortalecendo seu papel de defesa e educação em saúde pública.

## CONCLUSÕES

Há uma perceptível necessidade de medidas que visem atenuar o alto número de recusas familiares associadas à doação de pele. Nesse contexto, a identificação de características específicas das recusas pode configurar uma solução para esse problema. Como demonstrou a presente análise, é notório que doadores do sexo feminino e em faixas etárias mais avançadas tendem a ter seu tecido recusado, variando conforme o período analisado. Esses dados demonstram que outros fatores podem estar associados à recusa familiar de doação de pele, como o desconhecimento, os preconceitos e a baixa visibilidade em torno da temática, que impactam a tomada de decisão familiar.

Portanto, é imperativo aprofundar pesquisas na área e verificar dados como os do presente trabalho a fim de subsidiar ações e estratégias para efetivar um maior número de doações de pele e reduzir sua recusa. Os entraves sociais em torno do tema podem ser mitigados com medidas educativas vinculadas às universidades e aos grandes veículos de comunicação.
